# Awareness and control methods of gastrointestinal parasites of merino sheep among farmers from different agro-ecological zones of Lesotho

**DOI:** 10.14202/vetworld.2021.2554-2560

**Published:** 2021-09-27

**Authors:** Mots’elisi Aloycia Mahlehla, Setsumi Mots’oene Molapo, Mpho Wycliffe Phoofolo, Puleng Agathah Matebesi, Moeketsi Phalatsi, Morai Johannes Moiloa

**Affiliations:** 1 Department of Animal Sciences, National University of Lesotho, P.O. Roma 180, Lesotho; 2 Department of Biology, National University of Lesotho, P.O. Roma 180, Lesotho.

**Keywords:** farmers’, perspective, gastrointestinal parasites, Lesotho, Merino sheep farmers

## Abstract

**Background and Aim:**

Sheep production plays a crucial role in the economy of Lesotho by increasing economic state of the rural poor. However, gastrointestinal parasites infection is the most limiting factor in sheep productivity and has a highly detrimental effect on the sheep industry. Therefore this study aimed to evaluate farmers’ awareness and understanding of controlling gastrointestinal parasites of merino sheep in four Lesotho agro-ecological zones.

**Materials and Methods:**

Data were collected using a simple random sampling of 106 farmers in the lowlands, foothills, mountains, and Senqu river valley. Data were analyzed using the Statistical Package for the Social Sciences (version 20.00). Descriptive statistics were employed with cross-tabulations and tested with Chi-square and *post hoc* tests.

**Results:**

The majority of farmers (80%) were aware of the gastrointestinal parasites. Most farmers (70%) reported a high prevalence of gastrointestinal parasites and associated it with lamb mortality in summer and autumn. Farmers use anthelmintics for treating gastrointestinal parasites in animals, and 93% of them reported the effectiveness of the drugs. However, more than 80% of farmers routinely use anthelmintics. The results revealed that 81.5%, 66.7%, 80%, and 66.7% of farmers from the lowlands, foothills, mountains, and Senqu river valley, respectively, keep sheep in non-roofed enclosures cleaned only after rains to avoid mud. Communal grazing is used as the main source of animal feeding where different livestock species share the same rangelands. Most farmers (more than 70%) believed that grazing lands were the main source of gastrointestinal parasites transmission.

**Conclusion:**

Farmers in Lesotho are aware of gastrointestinal parasites and apply control methods to combat the gastrointestinal parasites in merino sheep. However, a need still exists for them to be empowered with skills for improving management systems and the knowledge on how the gastrointestinal parasites behave at different times of the year and in different agro-ecological zones. This will assist them in adhering to the dosing schedule designed by animal health experts.

## Introduction

Livestock farming assumes a critical role in subsistence agriculture in Lesotho, as in any other Sub-Saharan African country [1]. Livestock can increase income for most farmers, especially the rural resource poor and could serve as insurance against food deficit during extended droughts [2,3]. However, the health and productivity of grazing sheep are compromised due to the prevalence of gastrointestinal parasites that lead to higher use of anthelmintics [4]. Anthelmintics have proved successful in controlling different gastrointestinal parasites, especially when integrated with good farm management practices [5], although the effectiveness of anthelmintics is lessened by the development of drug resistance in many animals. This phenomenon has been a great problem for different regions in the world [6,7]. This problem is caused by the incorrect use of commercial/synthetic anthelmintics such as the lack of skills by farmers to manage or apply medication [8]. According to Tabuti *et al*. [9], the use of ethnic medicine to control gastrointestinal parasites is gaining popularity, especially in developing countries because of being readily accessible and easy preparation and administration.

Merino sheep farmers in Lesotho are faced with the challenge of high mortality rates, mostly seen in lambs, and decreased wool yield per sheep (average, 2.27 kg/year) [10]. Consequently, gastrointestinal tract parasites are highly implicated. Despite the use of commercial medication, mortality rates, in many cases, are not reduced which could be attributed to improper drugs administration. Sheep production is mainly in the hands of less educated and unskilled farmers [11], and this in itself is believed to be constrained in expanding sheep production in Lesotho.

Therefore, the objective of this study was to assess farmers’ understanding, perception, and animal management practices of gastrointestinal parasites in sheep as well as the control methods used.

## Materials and Methods

### Ethical approval

The ethical approval was granted by the Department of Animal Science of the National University of Lesotho.

### Study period and areas

The study was conducted from January to June 2016. The study was conducted in four agro-ecological zones (lowlands, foothills, mountains, and Senqu river valley) of the Maseru and Quthing districts in Lesotho. Each agro-ecological zone was represented by three villages sharing one woolshed. The lowlands were represented by Mahloenyeng-, Ha-Paanya-, and Morija villages, while the foothills were represented by Ha-Lebamang-, Ha-Chele-, and Thabana-Li-Mele villages. Both the lowlands and foothills are located in the Maseru district. The Senqu river valley was represented by Namolong-, Phokeng-, and Mapekeng villages, while the mountains were represented by Ha-Mohlakoana-, Matamong-, and Lebelonyana villages. These two agro-ecological zones (Senqu river valley and mountains) are located in Quthing district. The names of the woolsheds and the geographical coordinates of the areas where the research was conducted are displayed in Table-1.

**Table-1:** The agro-ecological zones, woolshed names, and their coordinates in the study area.

Agro-ecological zone	Woolshed	Coordinates
Lowlands	Matsieng	–29.617707, 27.555194
Foothills	Nyakosoba	–29.525359, 27.775831
Senqu river valley	Mount Moorosi	–30.281096, 27.858639
Mountains	Lebelonyana	–30.179263, 27.985847

### Sampling and data collection

A cross-sectional study was conducted using a purposive sampling procedure of 106 respondents engaged in oral interviews to complete questionnaires. A briefing and brainstorming session was held with officers from the Department of Livestock Services, Field Services, and Lesotho Wool and Mohair Growers Association on the objectives of the study and farmers’ selection criteria before the selection of farmers. Twenty-seven farmers per agro-ecological zone were randomly selected, and each village was represented by nine farmers except for the Senqu river valley and the mountains with eight farmers representing each village. The farmers were categorized into three groups according to sheep flock sizes per village to ensure that farmers of different flock sizes were represented. These are <50, <51-100, and >100 sheep representing small, medium, and large flock size categories, respectively. Three farmers, therefore, represented each flock size category.

### Statistical analysis

Data were analyzed using the Statistical Package for the Social Sciences (version 20.00, IBM, Armonk, NY, USA). Descriptive statistics were employed with cross-tabulations and tested with Chi-square test and Fisher’s exact test to assess the association of agro-ecological zones and the following characteristics: Farmers’ gender, education, and experience in the sheep industry and awareness, perception, control practices, sheep grazing, and management practices of farmers against gastrointestinal parasites. A *post hoc* test was employed to test the significance level (p=0.05) where any value below 0.05 was considered significant. Percentages, adjusted *z*-scores, and p-values were used to describe the farmers’ socioeconomic characteristics and management practices.

## Results

### Socioeconomic characteristics of farmers

The socioeconomic characteristics of the respondents are displayed in Table-2. The results of this study illustrated that male farmers constitute >80% of the farmers’ population in all the agro-ecological zones. Sheep farming was dominated by farmers possessing only primary education, and the majority of those farmers were found in the lowlands (74.10%). The highest numbers of farmers without formal education were found in the mountains (32%) and Senqu river valley (33.3%), compared with the other two zones where only 14.8% were illiterate. However, education levels do not differ statistically (p>0.05) across all agro-ecological zones.

**Table-2:** Farmers’ socioeconomic characteristics in the agro-ecological zones of the study area.

Profile	Category	Lowlands (%)	Foothills (%)	Mountains (%)	Senqu valley (%)
Gender	Male	81.50	85.20	84.00	96.30
	Female	18.50	14.80	16.00	3.70
Educational attainment	Illiterate	14.80	14.80	32.00	33.30
	Primary	74.10	55.60	40.00	59.30
	Secondary	7.40	18.50	16.00	3.70
	High school	3.70	7.40	12.00	0.00
	Tertiary	0.00	3.70	0.00	3.70
No formal education	Yes	44.40	48.10	40.00	66.70
	No	55.60	51.90	60.00	33.30
Experience	1-5 years	29.60	37.00	20.00	22.20
	6-10 years	11.10	37.00	20.00	22.20
	11-20 years	33.30	18.50	32.00	11.10
	>20 years	25.90	7.40	28.00	44.40

The population of farmers with ≥11 (categories, 11-20 and >20 years combined) years of sheep production experience was higher in the lowlands (59.20%), mountains (60%), and Senqu river valley (55.5%) than in the foothills (25.9%). However, more farmers with <10 years’ experience (categories 1-5 and 6-10 years combined) were noted in the foothills, possibly due to the number of young farmers joining sheep farming.

### Farmers’ perception on the impact of gastrointestinal parasites

**Figure-1:**
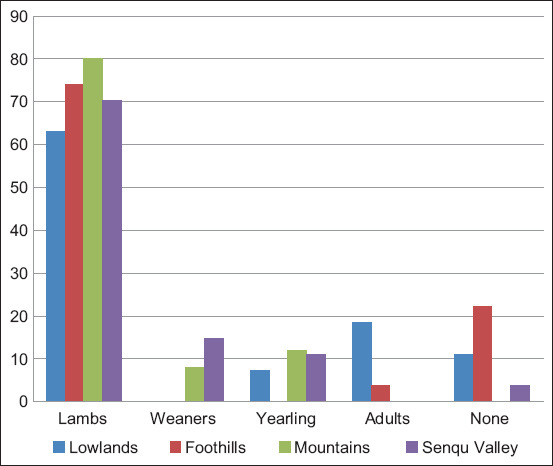
Mortality rates in different age groups of sheep in agro-ecological zones.

A significant (p<0.05) percentage of farmers (>80%) in all agro-ecological zones indicated that gastrointestinal parasites are a major problem in sheep farming leading to high mortality rates. Most farmers in the lowlands (63%), foothills (74.1%), mountains (80%), and Senqu river valley (70.4%) regarded lambs to be more vulnerable to mortality caused by gastrointestinal parasites compared with other age groups of sheep (Figure-1), and diarrhea is the foremost clinical symptom. The *post hoc* test indicated a significant association (18.5%) between adult sheep mortalities and the lowlands (*z*-score 3.30, p=0.00). A significant residual analysis was noted indicating that a higher than expected proportion of farmers reported that none of their sheep expired due to diarrhea in the lowlands (55.6%) and foothills (55.6%) at both *z*-scores of 3.60 (p=0.00). In addition, no animals expired because of diarrhea in the mountains while significantly (p<0.05) fewer numbers were reported in the Senqu river valley with a *z*-score of −3.80.

**Figure-2:**
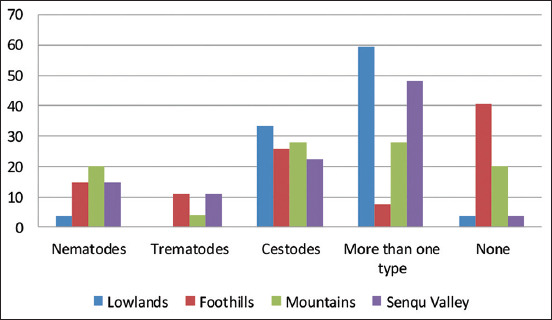
Farmers acquaintance to different gastrointestinal parasites in agro-ecological zones.

More than 90% of sheep farmers observed that their herds experienced high gastrointestinal parasites infections in summer and autumn. Stomach bots were reported by some sheep farmers (4%) in the lowlands, foothills, and mountains to be common in winter. Tapeworms were considered to be the most prevalent type of gastrointestinal parasites followed by nematodes (Figure-2). However, farmers from the lowlands reported that all gastrointestinal parasites are similarly prevalent (*z*-score=2.94, p=0.00). Unawareness of gastrointestinal parasites to farmers showed a significant association with the agro-ecological zone (*z*-score=3.80, p=0.00) with the residual analysis showing a higher than expected proportion of farmers in the foothills (40.7%) as well as the lower than expected proportion of foothill farmers (7.4%) who reported all gastrointestinal parasites to be significantly different (*z*-score=−3.60, p=0.00).

### Control practices

**Figure-3:**
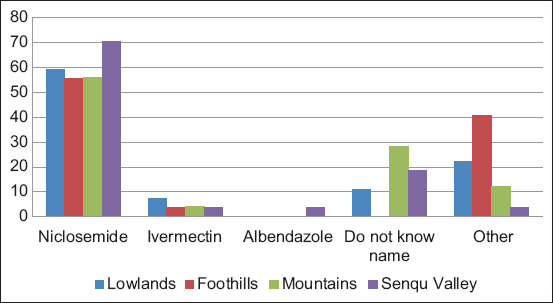
Anthelmintics used by farmers in different agroecological zones.

**Figure-4:**
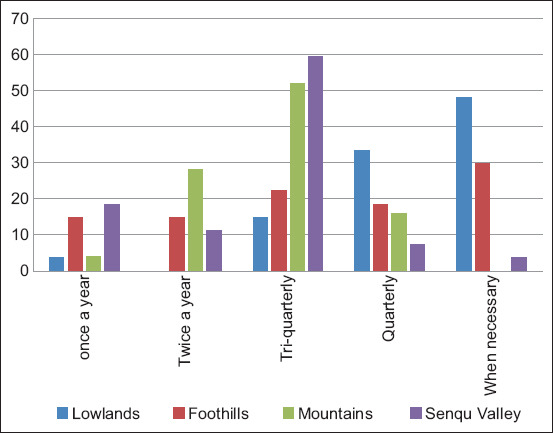
Frequency of anthelmintics use by farmers in different agro-ecological zones.

The study revealed that >90% of farmers regard the use of commercial anthelmintics as the most common method of controlling gastrointestinal parasites in all agro-ecological zones (Figure-3). The frequency of administering anthelmintics to sheep (Figure-4) varied greatly from farmer to farmer in different agro-ecological zones, although a higher than expected proportion of lowlands farmers reported a routine drug administration (48.00%; *z*-score=4.10, p=0.00). The use of niclosamide (Lintex, Germany) by farmers was >50% in all agro-ecological zones. Moreover, 40.70%, 59.30%, 48.0%, and 51.90% of farmers in the lowlands, foothills, mountains, and Senqu river valley, respectively, used ethnoveterinary medicine in addition to commercial anthelmintics with a significantly higher than expected proportion of lowlands farmers (40.7%) who mainly used ethnoveterinary medicine (*z*-score=3.20, p=0.00).

### Grazing and management practice

The majority of the respondents (≥80.00%) indicated that rangelands were the principal source of feeds for their animals (Table-3). However, 70.00% of farmers regarded rangelands as the major source of gastrointestinal parasite transmission because animals share the same rangelands (communal grazing). The majority of the farmers (70%) said that transmission of parasites is worsened by overgrazing of the rangelands due to overstocking and mixed livestock species on one rangeland.

**Table-3:** Grazing and feeding strategies of sheep farmers in different agro-ecological zones.

Category	Options	Lowlands	Foothills	Mountains	Senqu valley
Grazing area	Rangelands	85.20	92.60	80.00	96.30
	Pastures	14.80	7.40	12.00	3.70
	Fallowed fields	0.00	0.00	8.00	0.00
Villages with common rangeland	1-2 villages	29.60	33.30	20.00	11.10
	3-5 villages	48.10	40.70	28.00	51.90
	>5 villages	22.20	25.90	52.00	37.00
Grazing sufficiency	Yes	70.40	20.40	52.00	77.80
	No	29.60	29.60	48.00	22.20
Supplementation	Yes	77.80	88.90	92.00	74.10
	No	22.20	11.10	8.00	25.90

**Figure-5:**
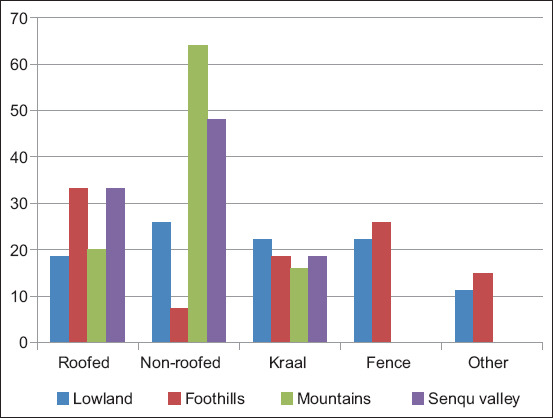
Housing types used by farmers in the agro-ecological zones.

The majority of farmers in the mountains (68.00%) and Senqu river valley (48.10%) housed their sheep in non-roofed kraals (Figure-5). The non-roofed enclosures were significantly associated with agro-ecological zones with the lower than expected proportion of foothill farmers (7.40%; *z*-score=3.60, p=0.00) as well as the higher than expected proportion of mountain farmers (64.00%; *z*-score=3.40, p=0.00). However, farmers in the lowlands (11.10%) and foothills (15%) keep their animals unconfined in an open space.

**Figure-6:**
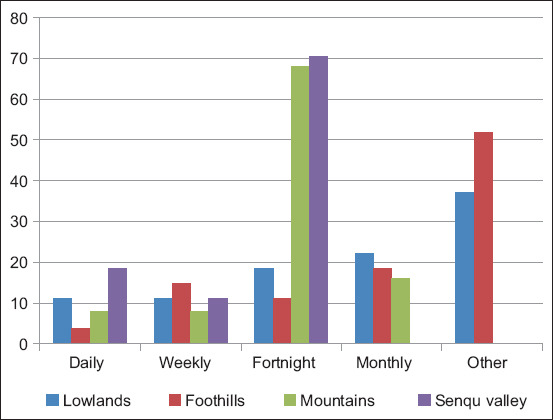
The frequency of cleaning animal enclosures.

The sheep farmers in the lowlands (37.00%) and foothills (51.90%) cleaned their animal enclosures only after rains to remove mud while 68.00% and 70.40% of farmers in the mountains and foothills, respectively, cleaned fortnightly (Figure-6). The *post hoc* test revealed a significant association of fortnight cleaning with the lower than expected (11.00%; *z*-score=−3.70, p=0.00) and higher than expected proportion of foothill farmers (51.90%) who reported cleaning only when muddy (*z*-score=4.20, p=0.00). A significantly higher than expected proportion of farmers (68%) reported cleaning animal housing fortnightly (*z*-score=3.10, p=0.00) and a significantly lower than expected proportion of farmers (0.00%) also cleaned animals housing fortnightly (*z*-score=−3.10, p=0.00) in the mountains. A significantly higher than expected proportion of farmers (70%) cleaned fortnightly (*z*-score=3.5, p=0.00) while a significantly lower than expected proportion of farmers (0.00%) also cleaned animals housing fortnightly (*z*-score=−3.3, p=0.00) in the Senqu river valley.

## Discussion

Sheep farming is a major source of livelihood in all agro-ecological zones and is in the hands of middle-aged male farmers in the lowlands. This observation appears to be in order because livestock farming activity is culturally perceived as more or less tedious work that needs a lot of energy and strength. This is supported by Adams and Yankyera [2], who stated that it is common in developing countries where men are culturally responsible for household productive assets than females. However, adopting newer production techniques may be a challenge because most farmers only possessed primary education (58%). The significance of education in farming is attested by Kaler and Rustonb [12] who emphasized that sheep farming improvements and technologies could be restrained without education. This underscores the need for training sheep farmers by strengthening extension services. The respondents regard gastrointestinal parasites as a major threat to sheep production and consider lambs to be severely affected. The susceptibility of sheep to internal parasites is caused by high sheep density and the ability of fecal pellets to disintegrate very easily, releasing the worm larvae on the pastures and rangelands, thereby enhancing the spread of gastrointestinal parasites [13]. Sheep become more vulnerable because they graze very close to the soil and easily acquire Stage 3 infective larvae and oocysts. This is further worsened by much rangeland degradation because of overgrazing and climate change, and the animals could easily acquire infective parasite stages. The severity of gastrointestinal parasites’ effects seen mainly in lambs is usually associated with high mortality rates. Some researchers [14] associated the mortality of lambs with severe diarrhea caused by coccidiosis and continued to mention that coccidiosis is generally seen in young animals because their immune systems have not developed the ability to combat heavy infections, which would, therefore, explain the high lamb mortality reported by farmers in this study. Lambs most probably are exposed to the parasites from their mothers during suckling dung-soiled teats [15], especially when farmers rarely clean their kraals and animals get in contact with old feces (Figure-6). Moreover, Hendawy, Alade and Bwala, [16,17] also indicated that the susceptibility of lambs to gastrointestinal parasites is also attributed to weaker immunological response because lambs have not been exposed to such infections before.

Controlling gastrointestinal parasites should not be a difficult task because farmers are aware of some gastrointestinal parasites, and a solution to a problem can only be found when the problem is known. Farmers in the agro-ecological zones considered that gastrointestinal parasites prevalence is a big challenge for sheep production in summer and autumn, but stomach bots attack animals in winter. The parasite (stomach bot) was initially known to be attacking horses but recently attacks even sheep [18]. This may be caused by communal grazing with mixed animal species grazing together, which allows cross-infection among different animals. The prevalence of gastrointestinal parasites varies depending on temperatures and rainfall patterns, as indicated by Shearer and Ezenwa [19]. This is also in line with Singh *et al*. [20] and Varadharajan and Vijayalakshmi [21], who indicated that variation in the prevalence of parasitic infestation depends on the differences in agroclimatic conditions and availability of a susceptible host. In addition, the results of this study agree greatly with Islam *et al*. [22], who indicated a higher prevalence of gastrointestinal parasites in warm/wet seasons.

Farmers admitted to administering anthelmintics to animals as a matter of routine (i.e., without seeing any sign or a predisposing factor to gastrointestinal parasites). Routine drug administration can result in underuse/underdosage or overuse/overdosage wherein both of which lead to resistance development of gastrointestinal parasites [23,24]. This may be attributed to the lower education level of the majority of the farmers, which enables inappropriate use of medications. Thus, some farmers complained that their animals still expired even after drug administration. Some farmers reported using numerous ethnoveterinary medicines to prevent mortality and improve livestock health. These medications have an apparent rationale and beneficial effects in many cases [25]. Ethnoveterinary medicine is gaining popularity in developing countries because it is readily accessible, easy to prepare and administer, and available at little or no cost to the farmer [26].

The majority of the farmers (>70%) considered communal grazing to be a serious problem in the management and control of animal diseases. In line with these results, Tsotetsi and Mbati [27] confirmed that communal grazing favors the development and scattering of gastrointestinal parasites. In addition, knowledge of life cycles and their timing is important in controlling parasites because certain drugs are only effective against specific stages in parasite development. Moreover, control is sometimes possible by reducing the number of intermediate hosts such as in tapeworms and liver flukes [28]. Under normal ­circumstances, the life cycle of most gastrointestinal parasites (nematodes, cestodes, and trematodes) generally takes about 3-4 months which the farmers at least should be aware of when doing rotational grazing in their rangelands. Rotational grazing could work best if done after 1-3 days post-deworming to allow sheep to move to clean grazing areas after shedding larvae and eggs at the previous grazing land. Controlling gastrointestinal parasites must be an integration of drug use and good grazing systems. Different animal species should graze at different times on different rangelands for maximum economic gain [29] to avoid accidental/incidental parasites transmission between species. Farmers agree that the different species of animals grazing together in one rangeland are a major contributory factor to animals having common gastrointestinal parasites.

Most farmers keeping sheep in structures that are not roofed, especially during cold seasons, could be one of the reasons for coccidial infection because of cold stress. This is supported by reports by Piirsalu *et al*. [30] and Food and Agricultural Organization [31], which indicated that improper sheep housing can be a bit of a problem because it may cause stress to animals. Lambs exposed to coldness could be susceptible to coccidial infection because of stress [32]. Furthermore, the failure of farmers to regularly clean their animal enclosures could lead to the accumulation of animals’ droppings which can harbor parasites eggs or even larval stages. Furthermore, transmission to suckling lambs could be easy because the possibility of suckling on the soiled teats cannot be avoided and, therefore, dams can be the source of transmission to the lambs as reported by Coffey and Hale [24] and Yakhchali and Zarei [32].

## Conclusion

The majority of sheep farmers in Lesotho have low education and skills. Hence, the practiced farming systems are more traditional. Farmers are aware of gastrointestinal parasites and use both anthelmintics and ethnoveterinary medicine to combat them. Among different age groups, lambs have comparatively high mortality rates. Most gastrointestinal parasites are prevalent in warm and wet seasons. Communal grazing is believed to be the main source of gastrointestinal transmission across all four agro-ecological zones. Most farmers keep their animals in open enclosures that are often uncleaned. Therefore, a need to train farmers on animal management practices and effective control measures aimed at controlling gastrointestinal parasites is recommended to improve the productivity of merino sheep in Lesotho.

## Authors’ Contributions

MAM and SMM: Participated in the data collection, analysis, preparation, and revision of the manuscript. MP and MJM: Involved in the collection of data and laboratory analysis. MWP and PAM: Conceptualized and designed the experiment. All authors read and approved the final manuscript.
